# The participation of lifeworld experts in Delphi processes: A reflection on method and practice

**DOI:** 10.1016/j.mex.2025.103274

**Published:** 2025-03-19

**Authors:** Marlen Niederberger, Marco Sonnberger

**Affiliations:** a PH Schwäbisch Gmünd, Institut für Gesundheitswissenschaften, Abt. Für Forschungsmethoden in der Gesundheitsförderung und Prävention, Oberbettringer Str. 200, Schwäbisch, Gmünd 73525, Germany; b University of Stuttgart, Department of Sociology of Technology, Risk and Environment (SOWI V), Seidenstrasse 36, Stuttgart 70174, Germany

**Keywords:** Delphi technique, Consensus, Participation, Health sciences, Patients, Review, *Delphi method*

## Abstract

Delphi studies have established themselves in the health sciences as a means to systematically and, ideally, synthesize expert opinions into a consensus on concrete issues. As participatory health research increases in relevance, lifeworld experts (e.g., patients, caregiving relatives) are being included in Delphi surveys and their opinions placed alongside those of professional and scientific experts. Looking at the theory and methodology, we discuss the opportunities and challenges concerning result quality and derive practical implications for conducting Delphi studies involving lifeworld experts alongside scientific and/or professional experts.

Delphi techniques are understood here to be social interaction processes whose outcomes are a result of the participating experts’ conscious, cognitive judgment processes, and also shaped by individual, situational and cultural factors. The more heterogeneous the expert panel, in particular when lifeworld experts are participating, the more these influences vary. Expert panel composition and how diversity is handled prove significant to Delphi study results.

Our argument is based on an in-depth analysis of a systematic review of Delphi studies with lifeworld experts. We found that the inclusion of lifeworld experts in Delphi studies usually occurs relatively unsystematically and, furthermore, that results are not analysed separately according to expert group, although there would be good reasons for this. We have oriented the reporting here on PRISMA. To enhance the outcomes of Delphi studies that incorporate lifeworld experts alongside scientific and/or professional experts, we put forward specific recommendations that address potential biases arising from the participation of lifeworld experts.

## Review Highlights

• The integration of lifeworld experts in a Delphi process influences the results of Delphi studies.
• Experts form judgments based not only on their knowledge, but also on different (epistemic) cultural conditioning and spheres of experience.
• In particular when lifeworld experts are involved, researchers must carefully consider and reflect on the “social” moment of a Delphi process as it pertains to the quality of the results.

## Graphical abstract

**Figure:**
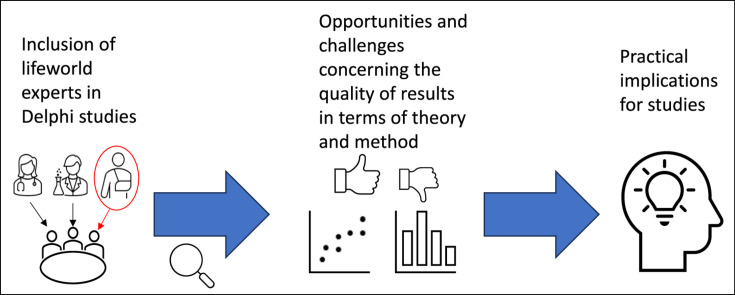

Specifications table

**Table:** 

Subject area:	*Nursing and Health Professions; Social Sciences*
More specific subject area:	*Participation of lifeworld experts in Delphi processes*
Name of the reviewed methodology:	*Delphi method*
Keywords:	*Delphi technique; consensus; participation; health sciences; patients; review*
Resource availability:	*DeWiss database of methods studies:*https://www.zotero.org/groups/4396781/dewiss_datenbanken_delphi-studien/collections/NGTBI3PE *DeWiss database of primary studies:*https://www.zotero.org/groups/4396781/dewiss_datenbanken_delphi-studien/collections/25H44TFI.
Review question:	*What methodological and practical aspects need to be taken into account when conducting Delphi studies with a heterogeneous group of experts that includes lifeworld experts?*

## Background

Delphi studies are used in a variety of ways in the health sciences (for different examples, see: [[1], [2], [3], [4]]). They are based on the assumption that a group of people with specific and complementary knowledge– experts–can assess and answer knowledge-based questions in such a way that yields a jointly supported and valid judgment which is more robust than isolated, individual judgments [5].

However, this assumption must be critically examined from an epistemic, methodological and practical perspective, for Delphi studies involve interactive group communication processes in which not just the rational consideration of facts and evidence, but also other aspects can influence the respondents’ judgments. Such factors are looked at in the following with critical reflection specifically on the participation of a heterogeneous expert panel in Delphi studies, which includes lifeworld experts. The **central question posed in this paper** is:

### What methodological and practical aspects need to be taken into account when conducting Delphi studies with a heterogeneous group of experts that includes lifeworld experts?

First, it must be clarified who can be viewed as an expert in the context of Delphi studies. These are people who possess bodies of knowledge that are of interest in connection with the object under investigation in a Delphi study. On an analytical level, it is possible to differentiate between **three groups of experts** while being fully aware that on a practical level many points of overlap are possible:

1. *Scientific experts*: These are understood to be experts who work at universities and research institutions, engage in research on the relevant topic and typically bring evidence-based knowledge to bear.
2. *Professional experts*: These are individuals who work in practical settings, for example, in the health professions, industry or administration. These experts primarily contribute implicit, non-codified knowledge, meaning knowledge that is inaccessible or difficult to access from the outside. This involves operational, functional, experiential and contextual knowledge [6,7].
3. *Lifeworld experts*: These are people who, due to life events or membership in a specific social community, have gathered concrete, real-world experience with the relevant topic and thus have acquired issue-specific knowledge based on individual experience [[8], [9], [10]]. For health-related topics, this can entail patients, caregivers or family members; in the case of environmental issues, this can mean the people who are affected by environmental problems. The main focus here is on practical everyday knowledge acquired through experience.

In Delphi surveys, scientific and professional experts are usually addressed as in their official function, and lifeworld experts as private persons [11]. While academic knowledge is highlighted in the case of scientific experts, for professional and lifeworld experts the focus is on their practical experience, whereby differentiation is made between these two groups regarding the basis of that experience. With professional experts this knowledge involves professionally acquired “objective” knowledge; by contrast, with lifeworld experts it is “subjective” experiential knowledge that comes from being directly affected. We do not presume any clear distinction here between the three groups, but rather assume that expertise can be placed on a continuum in terms of objectivity or subjectivity [12,13]. Ideally, scientific experts are characterized by an objective proximity to the relevant topic, whereas lifeworld experts distinguish themselves by having a subjective proximity based on direct personal experience. Positioned somewhere in between the two are the professional experts. All three groups are shaped by the different social contexts in which they are embedded and by the particular institutional or lifeworld practices and thought styles that prevail there.

We identify all three groups as experts because they are able to bring specific kinds of knowledge to bear in Delphi processes. We focus, however, on the specific challenges concerning the participation of lifeworld experts and place this group in opposition to the scientific and professional experts, while being fully aware that this represents a certain reductivity and distortion of the empirical reality.

In the following, we elucidate the basic principles of the Delphi method and draw from that the following hypothesis, as it relates to our main question: *The results of a Delphi study also depend on how heterogeneous the expert panel is, particularly in instances involving lifeworld experts, and how the interaction is designed as part of the research process.* In doing so, we will discuss a systematic literature review in which we have analysed the participation of lifeworld experts in Delphi studies. We will then turn our attention to the implications of our hypothesis in terms of practice.

## Method details

### The Delphi procedure: Concept and theoretical underpinnings

**Fig. 1:**
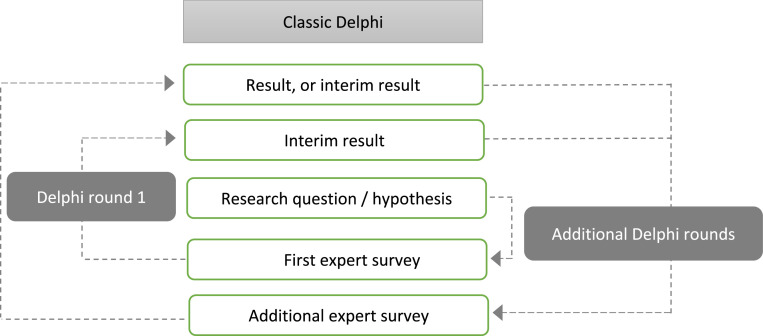
Procedure for a classic Delphi.

Delphi procedures are systematic, multi-step **group communication processes**, in which experts give their assessments and evaluations regarding a defined topic using a standardised questionnaire. What is special is that from the second Delphi round onwards the experts are able to see the aggregated results of the previous round and take this information into account as a corrective when forming their own judgments ([[14], [15], [16]]; Fig. 1). The goal is often to determine consensus regarding the relevant topic, meaning the maximum degree of convergence possible among the expert judgments or an agreement that no further convergence is possible, which is to say, consensus regarding dissent [17,18]. The procedure for a classic Delphi is illustrated in Fig. 1.

Typically, the methods literature on Delphi studies identifies **five central characteristics** [14,16]:

1. Experts are interviewed and their anonymity is preserved, at least during the Delphi process.
2. A standardised questionnaire is used.
3. Calculation of statistical group responses through univariate analysis.
4. Experts receive anonymized feedback with the option to revise their judgments.
5. One or several repetitions of the survey.

The **experts** generally participate based on the special knowledge attributed to them by the research team. Experts from academia and practice can be identified through publications or presentations, organisational memberships and field of work, or recommendations [12,19,20]. The aim is to include as much of the relevant expertise as possible in a balanced manner [21]. Systematic reviews of Delphi studies show that the number of experts who are surveyed ranges from less than ten to several thousand [22]. In an overview of systematic reviews of Delphi studies in the health sciences, Niederberger und Spranger [22] show that the average number of experts in the first round is 40 people.

There are now over ten different **Delphi variations** [23]. In addition to the classic procedure, there is also the real-time Delphi [24], the group Delphi [21], the policy Delphi [25], the spatial Delphi [26] and the argumentative Delphi [27]. These variations differ mainly in their focus on quantitative and qualitative elements and in regard to the intensity and immediacy of the interaction between the participants. Whereas closed questions are usually asked in the classic and real-time Delphi techniques and the quantification of the results is placed at the fore, the argumentative and policy Delphis are based more on open-ended qualitative questions. Furthermore, the group communication process is anonymous and asynchronous. Only in real-time Delphis are participants shown the aggregated responses in real time. Participants submit their judgments independently in both the synchronous and asynchronous variants. Only in group Delphis do the experts meet in workshop settings and respond to the survey questions in small rotating groups. Gaining qualitative and quantitative knowledge is the explicit aim of this variant. All Delphi variants, though, are **social interaction processes** in which, depending on the type of technique, the experts interact with each other synchronously or asynchronously, personally or anonymously, in order to contribute and consider their opinions.

**Fig. 2:**
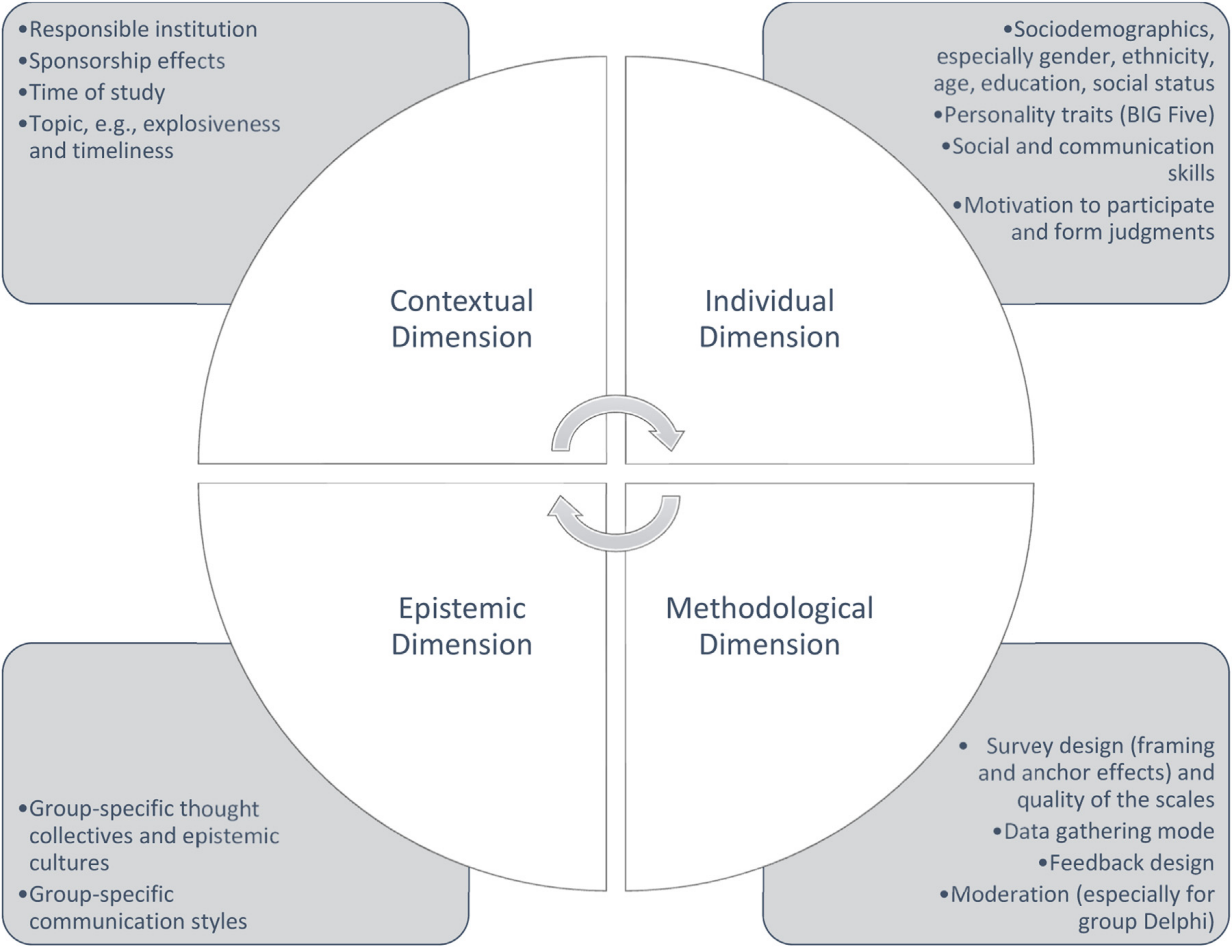
Important influences on judgment formation in Delphi processes (authors' illustration).

We assume that the outcome of a Delphi process is not solely the result of a rational, knowledge-based discourse, but rather, as **symbolic interactionism** argues, represents the result of a social interaction process within the scope of which meanings are not only reproduced, but also created [28]. The formation of such meanings is, in our view, influenced by the interplay of four dimensions (Fig. 2):

**Contextual dimension**: Here we refer to different conditions that can influence the Delphi process and therefore the result. Possible factors include the institution conducting the Delphi, sponsorship effects, the time point of the study and, in part connected to this, the currentness or contentiousness of the topic. It has been proven that these things can affect experts' motivation to participate in Delphi processes [29,30].

**Individual dimension**: This involves the individual characteristics of the participants such as gender, age, ethnicity, social status, social and communication skills, and educational or vocational background [[31], [32], [33]]. Also contained in this dimension, in our view, are the cognitive biases [34,35] that all people are subject to when making judgments but which can vary in the case of different personalities [36]. Likewise, the motivation to participate and form judgments also falls into this category, in respect to which the participants can differ from each other [31].

**Methodological dimension**: These are factors concerning the procedure of a Delphi study. Issues regarding questionnaire design and the wording of items, cognitive framing and anchors that are unintentionally applied as a result of process design, such as the type of moderation or the manner of giving feedback ([14,37,38], among others). For example, studies show that more lengthy and abstract statements make individual judgments more difficult and lead to more moderate opinions [37,39].

**Epistemic dimension**: To this dimension we assign group-specific forms of knowledge, notions of what is considered knowledge, and conventions regarding knowledge creation [40]. For example, representatives of different scientific disciplines belong to different epistemic cultures and cultivate different communication styles [41]. This obviously applies even more to participants from different social spheres such as, for instance, administration, business, civil society and the lifeworld. It would be possible to analyse this dimension by looking at the different judgment behaviours of expert groups; however, there are virtually no studies on this. The few analyses which do exist suggest that there are group effects [42,32].

In connection with this final point regarding the epistemic dimension, it must be assumed that new meanings are created especially when people who differ in their expertise [43] and belong to different epistemic cultures [41] are brought together in a Delphi process [12]. This coming together of different kinds of expertise and epistemic cultures causes irritation and friction that must be processed in the context of social interaction. From this perspective, the knowledge gained through Delphi techniques must, to a certain degree, always be viewed as group-specific and situational, since it has emerged from a specific social interaction process that, aside from all its structuredness, also contains situative and unique moments. Experiments have shown, for example, that in social interaction processes where differing opinions prevail group members move toward each other in their judgments and strengthen their self-confidence [44]. Hence, there is the risk in Delphi studies that convergence, and even consensus, is not based on substantial argument, but rather arises through social pressure [45], which in turn affirms the relevance of independent judgments.

It is clear that not only the result of a Delphi round, but also the entire interactive setting has an interactive effect on the participants and changes their cognitive and evaluative patterns of orientation in specific ways. Accordingly, participants in Delphi surveys frequently report a gain in their own competency or knowledge [12,46]. The outcome of a Delphi process is therefore not to be understood as just a convergence towards the “truth” from a positivist standpoint, but rather as a reconfiguration of the participating experts’ cognitive, evaluative and sociocultural patterns of orientation. Due to the social learning that occurs in the time and space of a specific Delphi process, the ensuing results must also be viewed as group-specific and situatively determined results, without the necessity to regard this as inherently negative or biased.

**The hypothesis** that we have drawn from these considerations and discuss in the following is: *The results of a Delphi study also depend on how heterogeneous the expert panel is, particularly in instances involving lifeworld experts, and how the interaction is designed as part of the research process.* The heterogeneity of the expert panel can have effects on different levels; in the following, though, we focus on synthesizing what is allegedly objective knowledge (scientific and professional experts) and subjective knowledge (lifeworld experts). The aim of the reflection is also meant to formulate practical recommendations on how Delphi practitioners can best design the inclusion of lifeworld experts.

Before we discuss the hypothesis from a theoretical standpoint, we would like to present the results of a systematic review. These findings demonstrate that the methodological handling of heterogeneous expert panels, including lifeworld experts, can vary widely and that no methodological criteria for the participation of lifeworld experts have yet been established for carrying out or assessing the quality of Delphi surveys.

### Participation of lifeworld experts in Delphi processes

Participatory research has clearly gained in relevance internationally in the health sciences. Currently, with the involvement of the International Collaboration for Participatory Health Research, its effects are being systematically and comparatively analysed and guidelines developed for conducting and evaluating such research projects [[47], [48], [49]]. The aim of participatory health research is to involve people with a specific lifeworld experience in research processes and, ideally, to give them a co-productive role throughout the research process [50]. They are approached as representatives of a community to which they feel they belong, for instance, due to health, cultural, geographic or political reasons [51]. They are usually reached through their activities in certain settings or their institutional memberships (e.g., hospitals, self-help groups, citizens’ initiatives).

All in all, the participation of lay people, referred to here as lifeworld experts, is increasingly becoming a quality marker of good health-related research. Patient involvement is called for especially in the development of medical guidelines and is now generally required in some countries, e.g., the UK [52,53]. The aims are to democratise the production of knowledge, take into consideration what is otherwise a “blind spot” in epistemological discourse and, ultimately, to generate valid, socially robust, applied knowledge. Accordingly, the participation of lifeworld experts in Delphi studies is gaining importance [[54], [55], [56], [57], [58], [59]]. They are involved in Delphi studies that centre on the development of core outcomes sets (COS) or concrete interactions with patients and their family members if they are immediately affected by the topic and their participation seems indispensable to successfully implementing the results into practice [60,61]. In the course of this, their judgments are gathered along with those of scientific and/or professional experts, although the lifeworld experts are usually in the minority according to their numbers compared to the other expert groups 56]. Despite this, differentiation according to expertise is not always undertaken when presenting the results of Delphi studies [54].

However, the initial differentiated analyses of actual Delphi studies indicate that when lifeworld experts participate, attention must first be paid to distinctive details in the conduction of the Delphi study and, second, that their participation affects the results [62,58]. Both of these aspects are present in an international Delphi study [63] focussed on developing consensus-based core outcome domains for trials in peritoneal dialysis (see Table 2 in the supplement). Here, two expert groups–patients/caregivers and members of the health professions–were surveyed anonymously in three online Delphi rounds. A total of 873 experts participated in the first Delphi round, of which 76 % were professional experts and 24 % lifeworld experts. The survey instruments contained explanations for the lifeworld experts so that they could understand statistical information and apply it to their judgements. The respondents had the opportunity in this Delphi procedure to record their judgments on a Likert scale and also justify them in open-ended texts. Clear differences were seen between the expert groups in their written justifications. Topics pertaining to extended treatment lengths, preserved quality of life, escalated morbidity, and futile interventions were more important for the patients/caregivers. In contrast to the health professionals, the patients/caregivers assigned higher priority to lifestyle-related outcomes and impacts on family or friends, while clinical aspects had more importance for the professional experts.

The Delphi study by Fauconnier et al. [64] on the development of a standardised questionnaire to measure symptoms of a specific disease also demonstrates that the expert groups respond differently (see Table 2 in the supplement). Clinicians and patients participated in this Delphi procedure. Two written Delphi rounds were conducted followed by a face-to-face meeting. Thirty-three patients and 23 clinicians participated in the first Delphi round, and three patients and seven clinicians attended the meeting. The results were presented in a differentiated manner according to the two expert groups. It was shown that the patients—the lifeworld experts—tended toward higher scale values, in effect, to a higher level of agreement than the clinicians on the written evaluation. The authors ultimately drew a positive conclusion in regard to the heterogeneity of the expert panel: “The DELPHI process, mixing different stakeholders, made it possible to select items that are easy to understand and represent the subjective experience of endometriosis, but that are also useful from a medical point of view” ([64], pp. 77).

Yet despite this, there is still no systematic overview of how lifeworld experts are integrated into Delphi studies. Such an overview is being compiled with the following systematic review.

### Systematic literature review on practical implications of the participation of lifeworld experts in Delphi processes

**Fig. 3:**
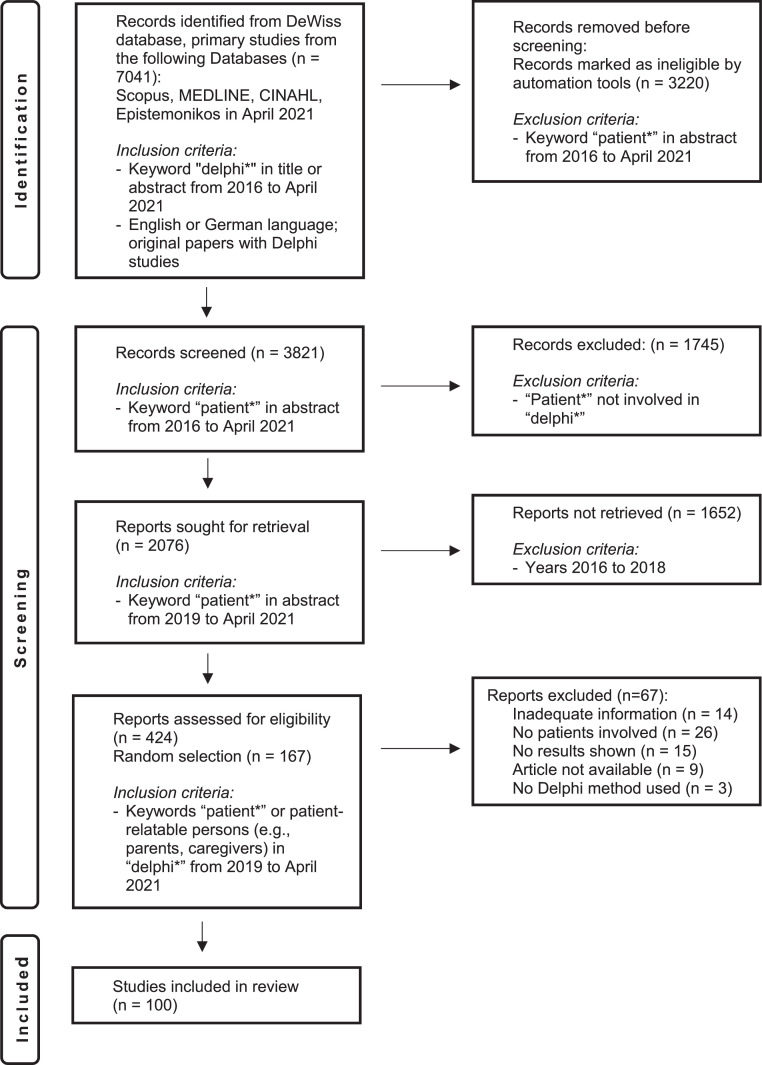
Flowchart showing the systematic literature review process (authors' illustration).

The goal of the systematic review is to capture Delphi practice regarding the inclusion of lifeworld experts (numbers in comparison to other expert groups, group-specific analysis, etc.). The basis of this review was the DeWiss database, freely accessible via Zotero, and which contains 7041 Delphi primary studies^1^ (for further information on the database, see [65]). These Delphi studies were in Scopus, MEDLINE, CINHAL und Epistemonikos. Once in the DeWiss database, all of the studies were screened using the search term “delphi*” in the title or abstract, with publication between 2016 and April 2021, in German or English. Following this, 2076 results were filtered out of the DeWiss database using the search term “patient*”, with a publication date between 2019 and April 2021. This search term served as an appropriate indicator of the potential inclusion of lifeworld experts in a Delphi study. The abstracts of each publication were read by two reviewers to ensure that these Delphi studies did indeed involve the inclusion of lifeworld experts. Excluded were Delphi publications in which patients were not part of the panel. This left 424 papers from which a random sample was taken with the ultimate aim of including 100 Delphi studies in the analysis (Fig. 3). We have oriented the reporting here on PRISMA (Page et al. [66]).

The analysis was carried out using deductive qualitative content analysis [67]. The following categories were defined:

1. Number of experts: total and group specific (scientific, practice, lifeworld);
2. Analysis and evaluation: type of feedback design and presentation of the results;
3. Effects: particular methodological details / published experiences regarding the inclusion of lifeworld experts.

The analysis using these categories enabled us to form a differentiated picture of how the inclusion of lifeworld experts is handled procedurally in different Delphi studies (categories 1 and 2) and whether specific effects or experiences are reported based on the inclusion of this expert group (category 3).

The first 20 publications were evaluated separately and independently by three reviewers. These results were then compared under the supervision of two Delphi experts (the authors of this paper), who, where necessary, looked again at the publications. Following this, a coding scheme was developed based on which the three reviewers went on to evaluate the rest of the publications.

The content analysis of the 100 publications shows that lifeworld experts in Delphi studies are in most cases underrepresented. In only 13 of the 100 Delphi studies analysed was the group of lifeworld experts and the other expert groups roughly equal in number [68]. Lifeworld experts were overrepresented in 11 studies [69], only lifeworld experts participated in 12 studies [70], and 10 studies reported no concrete frequencies [71]. In contrast to this, there were 54 Delphi studies in which the lifeworld experts were clearly underrepresented [72].

Furthermore, the results indicate that there is little interest in empirical practice regarding any differences in the judgments between the expert groups. In only 26 of the 100 Delphi studies are the results presented separately in the paper according to expert group [73]. And in only 14 of the 100 studies was feedback provided during the Delphi process based on the expert group [74]. In only eight Delphi studies did we find both the presentation of results separated according to expert group and group-specific feedback [75]. However, there were no reports that contributions of expert groups were weighted in order to eliminate the influence of a smaller numerical participation of any of the expert groups or that more specific statistical measures other than measures of central tendency, such as measures of dispersion, non-response rates or confidence levels, were applied to each expert group. We also found no indication that the differences between expert groups were actively discussed during the Delphi process among the participants. Furthermore, none of the Delphi studies discussed or reflected any differences in response behaviour.

On the whole, any specifics involving the inclusion of lifeworld experts, their judgments and weighting them in the research process hardly appear to be deliberately reflected in the empirical practice of Delphi techniques. In the following, we will discuss the implications this can have for the implementation and results of a Delphi process.

## Discussion

In the practice of Delphi studies, it seems in most cases that a balanced representation of the expert groups is not taken into consideration and when this is perhaps aimed for, it cannot be realized. The question arises here as to whether the originators of these Delphi studies assumed that there are no differences in judgment behaviour or when there were differences, deemed them irrelevant or not of relevance for publication.

This, however, contradicts the findings in the few Delphi studies that have already carried out differentiated analyses [64,63] (see section above), for the judgments and the response behaviours of the three expert groups differ [76]. Understandably so, lifeworld experts more often appear to prioritise aspects directly tangent to their lived environment. Scientific and professional experts appear to be more persistent in their judgments. Yet, as the results of the systematic review show, any such differences in judgment behaviour do not appear to be systematically scrutinized or reflected in the current practice and analysis of Delphi processes. It is to be assumed that the disclosure of the judgments according to expert group after the first Delphi round has an influence on response behaviour. The risk of bias exists as a consequence depending on group-specific disclosure of the judgments. However, insistence on the judgments of one's own expert group, ignoring the judgments of other expert groups, or the unconsidered consequences of judgements arrived at by other expert groups undermine the claim to a deliberate, knowledge-based discourse in Delphi procedures. When submitting judgments, cultural ideas, such as the “all-knowing scientist”, the “prejudiced practitioner”, the “aggrieved party” or the “egocentric patient” very likely play a certain role, whose effect thus far on Delphi processes remains completely opaque.

Given this context, it is also important to consider the epistemological principles of Delphi studies in order to gain a clearer perspective on the opportunities and risks of having a heterogeneous set of experts including lifeworld experts. Delphi techniques cannot be assigned solely to the positivist research paradigm nor to the constructivist research paradigm [[77], [78], [79]]. Instead, Delphi procedures alternate between the two epistemic perspectives:

1. **The positivist perspective**, according to which a Delphi process serves to move closer to an objective truth that is universally valid and therefore not bound to the set of participating experts. Other experts with comparable knowledge would have arrived at the same logical conclusion. The results of a Delphi process, then, must not only satisfy the quality criteria for validity but also those for reliability. A falsification of the results would only be possible in the case of new evidence-based knowledge. It is unknown to us whether or not this has ever been tested empirically.
2. **The constructivist perspective**, according to which a Delphi process represents a situative negotiation between the participating experts and therefore cannot be replicated one-to-one. The results of a Delphi process have validity and a certain meaning for the participants. The results are, however, to be viewed as situated results arrived at on the basis of a process bound by time and space. If a certain status, or even a political mandate, is ascribed to the participants, these results can also find acceptance and use, even if other experts, based on situational aspects, would have come to (somewhat) different judgments. According to this, Delphi processes must be evaluated according to quality criteria such as credibility, confirmability, transparency and reflexivity [80].

Which epistemological perspective appears more realistic depends significantly on the design of the survey instrument, including the weighting of the qualitative and quantitative elements, and—as hypothesized—on the composition of the expert panel. In the following are the epistemic and methodological hypotheses that, in our view, need to be investigated and discussed in further analyses:

• **The integration of lifeworld experts in a Delphi process influences the result of the Delphi study** [81]**.** This is why it is necessary to have a good balance between the three expert groups that can be described numerically and also reflected on the level of communication skills. Only if all of the expert groups equally understand the questions and the feedback in the second Delphi round onwards and are able to argue against and pass judgments on other judgments, including direct contradictions, will their perspectives be visible in the result. This is especially unlikely for lifeworld experts if they do not have the necessary formal educational background to understand the questions, if they hold back for reasons arising from the status and seniority of other expert groups, or if they perceive pressure to conform [82]. In these cases, there is the danger of “pseudo-participation” because their specific expertise will be lost in the typically descriptive presentation of Delphi results [83]. A positivist claim to knowledge would be tenable, at best, if the lifeworld experts were already able, or at least enabled during the Delphi process, to make and justify a judgment based primarily on cognitive aspects in order to eliminate situative or other controllable factors to the extent possible. We find that explicit training or additional, barrier-free information is conceivable here.
• **Experts do not judge based only on their knowledge, but also on different (epistemic) cultural conditioning and practices** (e.g., the definition of “consensus”, statistical practices). Accordingly, judgments are situated in specific epistemic cultures, a premise which stands in opposition to the claim of established universal truth. This is, according to the theoretical assumptions of epistemic cultures [41], important precisely in the case of divergent educational backgrounds and different discipline-specific affiliations [84]. It is possible that experts from another discipline or another specialty, but still belonging to the same expert group, would, for that reason, arrive at other judgments. A universal, positivist claim of validity for a Delphi result would only be possible if it is successful in keeping the conditioning of epistemic cultures as minimal as possible, for instance, through the most neutral phrasing possible or anonymous Delphi variations in which group-specific feedback is not communicated and discipline-specific practices in the analysis and feedback design are avoided. The extent to which this is even possible must be reflected and analysed in future Delphi studies.
• **The “social” moment in a Delphi process influences the way experts form judgments and hence the results**. From a positivist perspective, it is the “social” moment, more or less strongly influencing the results during the Delphi process depending on the technique, that appears to be a disruptive factor leading to biases [85]. For this reason, it is important to eliminate the social element to the extent possible and, in effect, avoid interactive Delphi variations or modifications and heterogeneous expert groups. Coming from a different angle, this seems impossible from a constructivist view, and an appropriately reflective approach is required [86].
• **Participation in the Delphi process has an effect on the participating experts. It sets individual empowerment processes in motion and shapes the social/professional identity** [48,87]. Empowerment processes are likely to occur particularly for lifeworld experts because they have the experience that their judgments are given the same significance and value as those of scientists and practitioners [62,51]. On the other hand, presenting the results separately according to expert group can raise awareness of lifeworld expertise among scientists and practitioners. As a consequence, the Delphi process, in turn, affects the context, whereby the dynamics and transformation are promoted. Hence, the claim of universal validity would be obsolete in the moment the result is arrived at This would only be possible in the case of Delphi surveys asking abstract or future-oriented questions.
• **In order to do justice to the diversity of lifeworld experts, creative methods adapted to the situation have to be applied** [53]: “Many themes reflect good practice in consumer engagement in guidelines, more broadly, but engaging with diverse groups may require greater attention to building formal, respectful partnerships and employing inclusive engagement. Both guideline organisations and funders have a role to play in creating a supportive environment” ([53]: 10). In our opinion, when applied to Delphi processes this means that, taking the methodological characteristics into account, modifications may be necessary in order to include lifeworld experts with different educational backgrounds and biographies on an equal basis. It is important that, even in the case of low numbers, their opinion does not get lost in the crowd and that they are enabled to submit, and even defend, a well-founded judgment on an equal footing with the other expert groups.

## Conclusion

As demonstrated in our review of Delphi studies incorporating lifeworld experts, their inclusion frequently occurs in relatively unconsidered and unspecific ways. The way in which the inclusion of lifeworld experts is reported in the papers is extremely heterogeneous and in part very sketchy. This implies that there is both a lack of appropriate standards and a prevailing need for appropriate reflection. Based on the argument laid out above, the following recommendations for research practice can be derived to improve the quality of Delphi studies including lifeworld experts. These recommendations are presented in Table 1. Whether these recommendations prove to be of value must undergo continued testing and reflection. A systematic comparison with other recommendations to ensure or improve the quality of Delphi procedures is also necessary [[88], [89], [90], [91], [92], [93], [94]].

**Table 1:** Practical recommendations for including lifeworld experts in Delphi processes.

Area	Practical advice
Expert panel composition	• Consider if the participation fundamentally makes sense and which blind spots are made visible. • During the entire Delphi process, emphasise its relevance and make it clear why each expert group is relevant.
Recruiting and dealing with different expert groups	• Watch for a certain balance among the group numbers when putting the expert panel together, and also across the different Delphi rounds. • If you offer an incentive to participate, fit this to match the particular needs or interests of each of the expert groups involved. • Give ongoing consideration to social group aspects, e.g., are all of the participants participating equally or not? • Make modifications to the Delphi process, as needed, taking the methodological characteristics into account in order to fully include lifeworld experts with different educational backgrounds and biographies (e.g., supporting materials or training on how to use the survey software or understand the statistics).
Delphi variants	• Given the topic/issue, consider which Delphi variant is suitable and where group differences are least effective in each case. • When including lifeworld experts, we advise using anonymous Delphi variants with clearly distinct rounds, so that, among other things, lifeworld experts also have sufficient time to respond. For this reason, the group Delphi and real-time Delphi appear less suitable.
Questionnaires	• Choose wording and phrasing for the questionnaire and any other materials (e.g., letters) that is understandable across disciplines and does not require academic study or specific technical knowledge. Information or trainings can also be made possible, as needed.
Analysis	• Carry out the analysis separately according to expert group (descriptive and inferential statistics) in each Delphi round and at the end and consider any differences to avoid biased results. • Consider weighting the results based on participant numbers in the expert groups so that even the numerically small groups are integrated equally.
Feedback design and presenting results	• When designing the feedback, avoid presenting the results according to expert group to prevent any undesired group effects (due to the authority bias, lifeworld experts in particular could align their assessments with those of scientific and professional experts in subsequent Delphi rounds). • Explain the results using statistical values and graphs.
Research team	• Include representatives of the lifeworld in the planning, conduction and evaluation of the Delphi process in order to become sensitized, if needed, to any specifics at an early stage.

These practical recommendations can also form a basis for developing quality criteria for a Delphi study planning to include lifeworld experts. Initial ways to assess the quality of patient involvement exist in the context of guideline development [52,95]. For example, van der Harm et al. [95] have developed a monitoring and evaluation framework with five topics and different indicators:

• Involvement of patients (e.g., balancing the number of patient representatives and professionals),
• Process structure (e.g., transparency of the process)
• Process management (e.g., facilitation of patient involvement)
• Direct outcomes (e.g., consensus on the content)
• Indirect outcomes (e.g., learning processes)

All in all, the result of a Delphi study depends significantly on how successfully contextual, individual, epistemic and methodological factors are taken into account. In regard to the validity and applicability of the findings, it is imperative to base the process on knowledge-based judgments and keep the influence of social and (epistemic) cultural factors to a minimum or, at least, to critically reflect on their effects. The need to include different expert groups in relatively equal proportions varies depending on the goals. In the case of research questions with practical implications, the inclusion of lifeworld experts appears to be more highly relevant than when determining potential future developments. A variety of challenges arise if the aim is to integrate lifeworld experts when conducting a Delphi study. Their participation assumes a certain level of personal involvement, skills and resources in advance. Only then do interactions on an equal level with other expert groups become likely. This requirement compounds the more open a Delphi process is. If there is personal and direct interaction between the expert groups, the risk of biased judgments increases as a result of status, seniority or personal competence. In the worst of cases, the participation of lifeworld experts would be pseudo-participation because their experiences would go unheard or would be lost in the dissent.

Participation in Delphi studies has previously been equated with integrating lifeworld experts into the expert panel. A broader understanding of participatory research with higher expectations could expand the reach of results obtained through Delphi studies. For instance, the inclusion of lifeworld experts in the research team is conceivable. As equal co-researchers, they could, for example, cooperate in developing the research design, implementing a Delphi study and presenting the results. Further potentials for knowledge could arise as a consequence.

We conclude the present paper with a concise discussion of methodological limitations. From our point of view, the empirical method of systematic review, which we have applied here, has four limitations:

• Only Delphi studies with patient involvement were included in the review. However, the participation of other groups of lifeworld experts is also conceivable (e.g., family members or friends). Nevertheless, we do not assume that dealing with other lifeworld expert groups is distinctly different from dealing with patients.
• The findings presented herein are derived from Delphi studies that have been published in academic journals. It is important to note that the criticisms articulated in this study may not be universally applicable to all Delphi studies, particularly those whose results are not disseminated publicly.
• It can be posited that the absence of reporting on certain aspects of the differences between expert groups in the published Delphi studies does not necessarily imply that these were not given consideration in the specific Delphi processes.
• Finally, it is crucial to acknowledge that the hypotheses derived and the practical recommendations formulated require empirical testing to substantiate their validity.

## Ethics statements

The author has followed MethodsX ethical guidelines, this work does not involve human subjects, animal experiments or data collected from social media.

## CRediT authorship contribution statement

**Marlen Niederberger:** Conceptualization, Methodology, Data curation, Formal analysis, Writing – original draft, Visualization, Investigation, Supervision, Writing – original draft. **Marco Sonnberger:** Conceptualization, Methodology, Data curation, Formal analysis, Writing – original draft, Visualization, Investigation, Writing – original draft.

## Declaration of competing interest

The authors declare that they have no known competing financial interests or personal relationships that could have appeared to influence the work reported in this paper.

## Footnotes

1 The DeWiss database can be accessed here: https://www.zotero.org/groups/4396781/dewiss_datenbanken_delphi-studien/library.
